# Assessing attention and impulsivity in the variable stimulus duration and variable intertrial interval rodent continuous performance test schedules using dopamine receptor antagonists in female C57BL/6JRj mice

**DOI:** 10.1007/s00213-023-06387-7

**Published:** 2023-06-28

**Authors:** L. Klem, M. M. Nielsen, S. B. Gestsdóttir, S. L. Frandsen, S. Prichardt, J. T. Andreasen

**Affiliations:** Department of Drug Design and Pharmacology, University of Copenhagen, Universitetsparken 2, 2100 Copenhagen, Denmark

**Keywords:** Attention, Impulsivity, Rodent continuous performance test, Dopamine, Antagonist, Arousal

## Abstract

**Rationale:**

Dopaminergic dysfunction is implicated in disorders of impulsivity and inattention. The rodent continuous performance test (rCPT) has been used to quantify changes in attention and impulsivity.

**Objective:**

To examine the roles of dopamine receptors in attention and impulsivity behaviours measured in the rCPT variable stimulus duration (vSD) and the variable intertrial interval schedules (vITI) using DA receptor antagonists.

**Methods:**

Two cohorts of 35 and 36 female C57BL/6JRj mice were examined separately in the rCPT, vSD, and vITI schedules, respectively. Both cohorts received antagonists of the following receptors: D_1/5_ (SCH23390, SCH: 0.01, 0.02, 0.04 mg/kg) and D_2/3_ (raclopride, RAC 0.03, 0.10, 0.30 mg/kg) in consecutive balanced Latin square designs with flanking reference measurements. The antagonists were subsequently examined for effects on locomotor activity.

**Results:**

SCH showed similar effects in both schedules, and the effects were reference-dependent in the vITI schedule. SCH reduced responding, but improved response accuracy, impulsivity, discriminability, and locomotor activity. RAC showed mixed effects on responsivity, but improved accuracy and discriminability. The discriminability improvement was driven by an increase in hit rate in the vITI schedule and a reduction in false alarm rate in the vSD schedule. RAC also decreased locomotor activity.

**Conclusion:**

Both D_1/5_ and D_2/3_ receptor antagonism reduced responding, but the outcome on discriminability differed, stemming from individual effects on hit and false alarm rate, and the weight of omissions within the calculation. The effects of SCH and RAC suggest that endogenous DA increases responding and impulsivity, but reduces accuracy and shows mixed effects on discriminability.

**Supplementary Information:**

The online version contains supplementary material available at 10.1007/s00213-023-06387-7.

## Introduction

Dopamine (DA) and noradrenaline (NA) are critically involved in regulating executive function, including attention and inhibitory control. Dysfunction of these catecholamine systems is associated with pathological inattention and impulsivity, symptoms that occur in, e.g., attention-deficit/hyperactivity disorder (ADHD), major depressive disorder, schizophrenia, and in Parkinson’s disease (Sonuga-Barke et al. 2010; Del Campo et al. 2011; Bluschke et al. 2017; Wolfers et al. 2020). Enhancement of catecholamine transmission through inhibition of DA and NA re-uptake effectively improves inattention and is the most common pharmacological treatment strategy for individuals with ADHD. The relationship between NA/DA transmission levels and attention performance has been described as an inverted U-shaped arousal-performance relationship, where both insufficient and excessive arousal are detrimental to performance (Arnsten et al. 2007; Arnsten and Robbins 2009). The prefrontal cortex (PFC) processes stimuli as relevant (signal) or discarded as irrelevant distractors (noise). DA and NA fine-tune the signal-to-noise ratio through different mechanisms, as NA facilitates coding of relevant stimuli, and DA facilitates removal of noise (Arnsten et al. 2007). The ability of DA to reduce noise involves activation of D_1_-like receptors, with improved performance at low-to-optimum DA levels, but impaired performance at higher DA levels, e.g., during stress. The outcome of D_1/5_ receptor activation or inhibition therefore depends on pre-existing DA levels (Phillips, 2004; Ramos and Arnsten, 2007; Vijayraghavan et al. 2007).

Continuous performance tests (CPT) assess attention and impulsivity in humans, and different rodent variants have been developed, including the rodent CPT (rCPT) (Kim et al. 2015). The rCPT presents stimuli in a single fixed location, similar to the human CPTs, while the 5-choice continuous performance test (5C-CPT) is an earlier rodent variant, in which the target stimulus is an illumination of one of five light apertures in a spatial array (Young et al. 2009), i.e. based on the 5-choice serial reaction time task (5-CSRTT) (Bari et al. 2008). Human and rodent CPTs offer paradigms to challenge different behaviours, including variable intertrial interval (vITI) and variable stimulus duration (vSD) schedules, and the rCPT is suitable for these paradigms (Kim et al. 2015). 5-CSRTT research has previously used vITI schedules to increase demand on inhibition of inappropriate responding, measured through increased premature responses (Robbins, 2002; Bari et al. 2008; Amitai and Markou, 2011; Callahan et al. 2019), while the vSD schedule is predominantly used to increase attentional demand measured as decreases in accuracy (Bari et al. 2008; Higgins and Breysse, 2008; Callahan et al. 2019). Using a fixed location for target (S+) and non-target (S−) stimuli presentation may improve translatability to human CPTs, but the assay permutations differ in the S+/S− presentation ratio. The rCPT schedules used in this study present both stimuli at an even 1:1 ratio, while human permutations typically deliver either mostly S+ (e.g., Conners’ CPT; 9:1 S+:S−) or S− stimuli (e.g., X-CPT; 1:5 S+:S−) to bias subjects towards responses (go) or non-responses (no-go), respectively. By not inducing this response bias, there is a lower cognitive demand on rodents in the rCPT relative to humans in the CPTs, e.g., as there is a less pre-potent no-go response. However, rCPTs must use a relatively higher S+ ratio than human CPTs, since task engagement of mice is driven by reward and that mice are not inherently motivated to engage in the task. By having an even 1:1 stimulo ratio, the rCPT more closely resembles the rodent go/no-go tasks than clinical CPTs (Kim et al. 2015). However, some human CPT studies used an even 1:1 probability (Losier et al. 1996), and it has been shown that high S+ probabilities (e.g., 50%) promote a more liberal response strategy, challenging inhibitory control systems (Lynn and Barrett, 2014).

We recently characterized the rCPT using selective and non-selective DA/NA reuptake inhibitors in male and female mice, using a vSD schedule (Caballero-Puntiverio et al. 2019, 2020). These drugs showed different effects by virtue of their different pharmacological profiles, but they all revealed prominent roles of catecholamines in regulating different behaviours measured in the rCPT. By including reference measurements of each test subject we found that the effects were largely reference-dependent, generally showing a larger improvement in attentional performance in mice with lower reference performances (Caballero-Puntiverio et al. 2020). The reference-dependent drug effects supported the relationship between attentional performance measured by the rCPT and the inverted U-shaped arousal-performance relationship.

To optimise the sensitivity towards impulsivity-modulating treatment effects, we modified a rCPT vITI schedule and separated the analysis of premature responses (PR) into the first touches (FiT, corresponding to premature responses in the 5-CSRTT and 5C-CPT) and following touches (FoT). Similar to the vSD schedule, we tested selective and non-selective DA/NA reuptake-inhibitors in the vITI schedule and found differential effects of NAT and DAT inhibition on PR (Prichardt et al. 2023).

The purpose of the present study was to examine the role of specific catecholamine receptor subtypes in the regulation of attention- and impulsivity-related behaviours in the rCPT. To this end, we tested antagonists at various NA and DA receptor types: the adrenoceptor antagonists doxazosin (α_1_), yohimbine (α_2_) and propranolol (β_1_), and the dopamine receptor antagonists SCH23390 (D_1/5_) and raclopride (D_2/3_). We employed both the vSD and the vITI schedules, to obtain maximal information about both attention- and impulsivity-related measures. Due to the large amount of data, the results are presented in two separate articles, showing the results of DA in this current article, and the NA results in a separate article (Klem et al. 2023).

## Materials and methods

We examined two cohorts of 35 and 36 female C57BL/6JRj mice (Janvier), which were 7 weeks old upon handling and ~10 months upon study completion. The mice were housed in groups of four per cage in a controlled environment with a relative humidity of 40–60%, at 20–22°C, and in a 12h/12h dark-light cycle with lights on at 8 AM. The cages were supplied with enrichment in the form of wooden sticks, climbing ropes, and red plastic shelters. The mice were habituated to the experimenter and liquid reinforcement (Yazoo Kids no added sugar strawberry milk) 1 week prior to training. The mice had free access to water and were maintained on a restricted feeding regimen down to 85% of their ad lib weight based on available growth curves from Janvier, to ensure adequate motivation and participation in the test. We housed female mice without male mice in the room, which may lead to the Lee-Boot effect, i.e., suppression or prolongation of the estrus cycle when females are housed together in isolation from males (Champlin 1971; Van Der Lee and Boot 1955). This practice of exclusively housing female mice results in lower levels of fighting or stress compared to males (Fredericson 1952; Scott and Fredericson 1951), contributing to a more ethical laboratory housing practice. Furthermore, the experiments were conducted in the lights phase, as our in-house rCPT data suggests that there is a limited impact of light/dark cycle on performance. This observation is supported by a comprehensive review, which found that mice excibited similar social scores in light and dark phases and that testing in the light phase adequately estimates those obtained in the dark phase (Yang et al. 2008). All procedures were approved by the Danish Animal Experiment Inspectorate, license no: 2017-15-0201-01195, and conducted in accordance with EU Directive 2010/63/EU and Directive 86/609/EEC for animal experiments.

### The rodent continuous performance test (rCPT)

The full study examined antagonism of NA adrenoceptors and DA receptors. Due to the extent of the collected data, the NA results are presented in a separate article (Klem et al. 2023) and the DA results presented here. For elaborate description of the rCPT training process, vSD and vITI setup, data transformation, statistical analysis, and explanation of the line graphs, please refer to the NA article (Klem et al. 2023). The following sections provide similar information in brief.

#### rCPT apparatus

The rCPT training and testing occurred in touchscreen operant trapezoid-like chambers in sound- and light-attenuated boxes (Campden Instruments Ltd., Leicester, UK). The front of the chambers had a touch-sensitive screen covered by a black acrylic mask with three identical cut-outs, and the visual stimuli were presented in the centre cut-out. The back of the chamber had a reward delivery magazine from where mice could retrieve the liquid reinforcer. When the mouse touched the target stimulus, a 1-s tone was evoked, the reward magazine was illuminated, and 20 μL strawberry milk (Yazoo, no added sugar) was delivered to the reward tray by a peristaltic pump. All the chamber operations were controlled by Whisker server (Cambridge, UK) and ABET II (Lafayette Instruments, Indianapolis, USA), which recorded and collected the raw data for further analysis. The experimenters were not blinded to the dosing regimen.

#### rCPT response types, flow, and parameters

Target (S+) and non-target (S−) stimuli are presented as a block-randomization at an even frequency (50%). During S+ presentation, a mouse can make a response by touching the screen (a hit) or an incorrectly withheld response (a miss). A hit evokes a 1-s tone and a 20 μL strawberry milk delivery. Touching the screen during an S− is defined as a false alarm (FA, also known as a ‘mistake’), while withholding a response is defined as a correct rejection. A FA triggers a correction trial in which another S− is displayed (a correction trial). This continues in a loop until the mouse correctly withholds S− responding. Note that correction trial mistakes and correct rejections are not included in the parameters calculated below. The next trial will then be regular trial, i.e., with 50% chance of an S+. A blank dark screen is shown between trials, i.e. during the inter-trial intervals (ITIs). ITIs varied according to the test schedule: in the vSD schedule, the ITI was either 2 or 3 s (50% of each), whereas the ITIs in the vITI schedule were 3, 6, or 12 s. Touches to the blank screen during the ITIs are recorded as premature responses and will restart the same ITI type. This so-called ITI correction loop continues until the mouse withholds responses to the blank screen for the entire ITI, and before the next trial can begin. In the standard schedule, these ITI correction restarts are a part of the trial in which the initial premature touch was made. In the vITI schedule premature responses (PR) are divided into first touches and following touches (Prichardt et al. 2023). The rCPT parameters are calculated as follows:

(i) Hit rate: $$HR=\frac{Hits+1}{Hits+ Misses+1}$$
(ii) False alarm rate: $FAR=\frac{Mistakes+1}{Mistakes+1+ Correct\ rejections}$
(iii)  Discriminability: $${d}^{\prime }=z(HR)-z(FAR)$$ 
(iv) Accuracy level: $\% Acc=100\%\ast \frac{Hits+1}{Hits+ Mistakes+1}$
(v) Response criterion $$:C=\frac{-\left(z(HR)+z(FAR)\right)}{2}$$
(vi) Premature response level: %PR $=\dfrac{Centre\ touches\ast 100\%}{Centre\ touches+ Total\ number\ of\ ITIs}$  
(vii) First touches level: $\% FiT=\dfrac{Initial\ centre\ touches\ during\ 12s\ ITI\ast 100\%}{Total\ number\ of\ 12s\ ITI s}$  
(viii) Ratio of following to first touches: $\dfrac{FoT}{FiT}=\dfrac{Centre\ touches\ during\ 12s\ ITI\ restart\ loop}{Initial\ centre\ touches\ during\ 12s\ ITI}$  

The vSD schedule assesses premature responses in the %PR parameter, while %FiT and FoT/FiT have replaced this parameter for the vITI schedule (Prichardt et al. 2023). Our previous study indicated that %FiT is a more sensitive measure of waiting impulsivity, while the FoT/FiT ratio remains to be characterised. To support this characterisation, we present the FoT/FiT results in the Supplementary information for our current DA study and for the related NA study (Klem et al. 2023, Supplementary information). In both our current and NA study, we include the accuracy parameter to bridge the gap between 5-CSRTT, 5C-CPT, and rCPT research (Klem et al. 2023).

#### rCPT training and task manipulations during testing

The rCPT training of these cohorts has been described in our previous work (Klem et al. 2023) and will be briefly described here. Table 1 shows a general overview of the setup for the final training stage and the two testing schedules. Training includes a chamber habituation session followed by four training stages of increasing difficulty, requiring a pre-defined discriminability between the presented stimuli. In the final training stage, the mice were required to discriminate between the S+ and the four S− stimuli. In each trial there was 50% chance of an S+ stimulus and 50% chance of one of the four S− stimuli. The S+ was counterbalanced across all mice as either horizontal or vertical lines, while the same four S- stimuli were used for all mice. The S− images included two different images with diagonal lines, a spiral, and either horizontal or vertical lined image that is orthogonal to the S+ stimulus. During the four training stages the stimulus duration was gradually reduced to 2.0 s, while the ITI was 2–3 s in all training stages. The limited hold is triggered upon stimulus presentation and defined as the time period, in which the screen will record touches. The limited hold was gradually reduced to 2.5 s, i.e. 0.5 s beyond the stimulus duration. The after-reward pause was fixed at 2.0 s for all trainings and testing schedules. The mice were given a daily session on weekdays, i.e. five sessions per week, and each session ran for 30 min or until the maximum number of rewards were achieved (100 for stages 1–2, and 150 for stages 3–4). Mice completed their training by passing stage 4, which required a minimum of seven sessions and a d’>1 for the two most recent sessions. After mice had completed stage 4, they were maintenance trained once weekly until testing began. The vSD schedule cohort received an average of 21 ± 3 (SD) sessions to complete training, while the vITI schedule cohort received 21 ± 5 sessions on average.

**Table 1:**
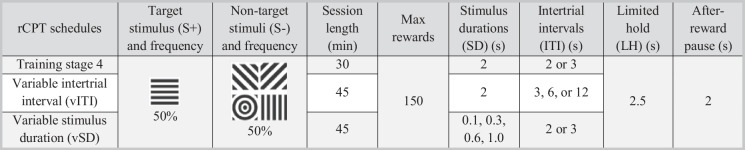
An overview of rodent continuous performance test (rCPT) schedule properties, comparing the final training stage with the two test schedules, exemplified for mice with horizontal lines as the target stimuli. Based on (Kim et al. 2015; Caballero-Puntiverio et al. 2020)

Following training, the mice were habituated to the allocated schedule (vSD or vITI) over the course of five sessions. Subcutaneously injections of 10 mL/kg 0.9% sodium chloride were given before each session to ensure sufficient habituation to the injection process prior to on testing. The vSD and vITI schedules have a setup similar to the final training stage, except the session length, which was extended to 45 min in the testing phase. The vITI schedule differed by including variable ITIs of 3, 6, or 12 s, which were used to increase the sampling duration for impulsive responding, while maintaining an unpredictable nature of the ITIs. The vSD used variable stimulus durations of 0.1, 0.3, 0.6, and 1.0 s to increase task difficulty and challenge attention. Despite the shorter stimulus duration, the 2.5 s limited hold provided the mice with the same amount of time to respond. Testing occurred twice weekly with a minimum of 3 days between sessions. The mice performed a baseline (stage 4) session on the day prior to testing to check for normal responding (d’>1). The drug studies were carried out as separate balanced Latin square designs (LSDs) flanked by two vehicle measurements, using the relevant schedule (vSD or vITI). The average value of the two flanking measurements was taken as the reference value of each animal. The LSDs included a vehicle measurement and measurements of three different doses of the antagonists. Drug-induced effects were expressed relative to the vehicle measurement value *within* the LSD, which was defined as 0. All compounds were administered subcutaneously in a volume of 10 mL/kg, 30 min prior to testing.

#### rCPT data transformation and analysis

The raw data was compiled using ABET software, and the parameters were calculated in Excel as described. As described in detail in Klem et al. (2023), data were transformed to comply with the assumptions of a parametric data analysis. Specifically, HR, FAR, %Acc, %FiT, and %PR were transformed using logit transformation, calculated as $\mathit{\ln}\left(\frac{Y- Lower\ limit}{Upper\ limit-Y}\right)$, where Y is the observed value, and lower and upper limits represent the lowest and highest theoretically possible value, respectively. By adding weight to values approaching the upper or lower limits, logit transformation accounts for floor and ceiling effects and thereby provide more valid measurements of dose:reference interactions (Klem et al. 2023, Supplementary information). The parameters d’ and C are based on Z-score calculations making them unconstrained by ceiling or floor effects; hence they were not transformed. We analysed the data in a repeated measurements mixed effect model, using the fitlme function, as initially presented by Caballero-Puntiverio et al. (2020). The statistical analyses were performed in MATLAB (Natick, MA, USA, version 2020b), containing the following model:

$$Parameter\sim 1+ dose+ time+ reference+ dose: reference+\left(1\ |\ animal\ ID\right)$$

Each parameter depends on several fixed effects as well as on the random effect represented as the animal ID and depicting the animal-to-animal variation. The intercept is included through the initial “1” in the formula. The main effects of treatment (also termed dose), time, and reference performance were examined. The dose:reference interaction was included to ascertain if treatment effects depended on the reference level of the animal. The dose:reference interaction was ascertained for the overall drug treatment for each drug as well as for the individual doses. The interaction was excluded from the model when the post hoc analysis showed non-significant results. The significance calculations for the different terms in the models were based on *F*-tests, and the individual fixed effect estimates were examined using *t*-tests.

We present our data in two formats: as the non-transformed values in bar charts, and as the logit-transformed data in the line charts, which are expressed as the transformed values used in statistical modelling. The bar charts were prepared using GraphPad Prism (version 9, La Jolla, California, USA) and include the means ± the standard errors of the means (SEM). The line graphs were prepared using MATLAB 2020b and depict the modelled data. The line graph Y-axis indicates the drug-induced effects relative to the internal LSD vehicle value, which is shown as Y=0 and represents the individual vehicle response value for each mouse. The line graph X-axis arranges the performance of the mice according to their reference values (i.e., the average of the response to the two vehicle treatments obtained before and after the LSD, respectively), and the mean reference value for the cohort is defined as X=0. This data analysis and presentation allows for detection of the overall dose effects as reflected by the value where the line intercepts with the dotted line at X=0, and the dose:reference interaction is reflected by the slope of the line. Asterisks were used to describe significant dose effects, *, whereas significant slope effects used hashtags, #. Results were considered significant for *P*-values below 0.05, with significance presented as follows: *P*<0.05*, *P*<0.01**, *P*<0.001***. Trend values, *P*<0.1(*), were reported in parenthesis. The validity of the statistical approach and test-retest reliability were analysed in correlation analyses of the reference values, shown in the supplementary information for the NA antagonist study Klem et al. 2023).

The cohort size of 36 mice was based on previous research within our group using the line graph analysis approach, which generally requires a minimum of 30 mice to ensure that a straight line can be drawn from the mice with low values on the one end of the x-axis towards the mice with high reference values on the other end of the scale (Caballero-Puntiverio et al. 2020, Prichardt et al. 2023).

### Locomotor assay setup and analysis

A locomotor activity assay was included to assess for non-specific motor effects of the antagonists, which may confound the interpretation of the rCPT results. The tests were conducted in a dimply lit room in transparent type III H cages (L × W × H: 42.5 × 26.5 × 18 cm) on a white background. We covered the cage with plastic wrap with a few airholes to discourage jumping and escaping. The output was recorded by a camera mounted in the ceiling and coupled to a computer with EthoVision XT (Noldus) software (version 8). Testing began 5 min after positioning a mouse in the test cage, and the activity was recorded for 40 min, after which the mice were removed and the cages were cleaned between mice. We pooled the cohorts and ran two experiments separated by a 7-day washout period. The doses were randomized among the mice, but we ensured that all mice received different treatments. We compiled the raw data using EthoVision XT (Noldus) software. The total travelled distance was analysed through a one-way analysis of variance (ANOVA) with multiple comparisons relative to the vehicle (Dunnett’s). We presented means and standard errors of the means in bar charts using GraphPad Prism (version 9, La Jolla, California, USA). The results were considered significant for: *P*<0.05*, *P*<0.01**, *P*<0.001***. Trend values, *P*<0.1(*), were reported in parenthesis. The bar charts depict the observed data, while the statistical analysis was done on the log-transformed data.

### Pharmacological interventions

SCH23390 hydrochloride (SCH 0.01, 0.02, and 0.04 mg/kg) was purchased from Adooq. Raclopride tartrate (RAC 0.03, 0.10, 0.30 mg/kg) was purchased from Sigma Aldrich. The drugs were dissolved in a vehicle of 0.4 % dimethyl sulfoxide and 0.9% sodium chloride, and adjusted to pH = 7±1. The dose-ranges used in the studies were based on a literature review and on pilot dose-finding studies described in the supplementary information for the NA adrenoceptor article (Klem et al. 2023).

## Results

**Fig. 1:**
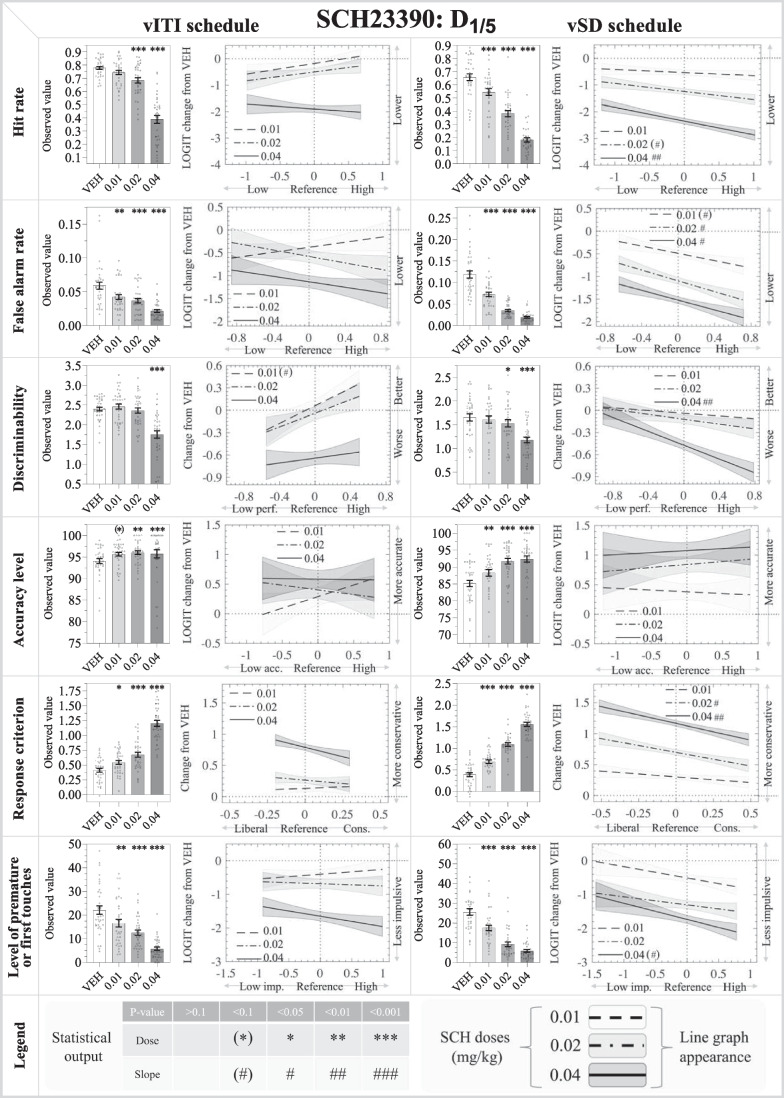
Results from the D_1/5_ receptor antagonist: SCH23390 (SCH: 0.01, 0.02, 0.04 mg/kg) in the rodent continuous performance test. The left- and right-hand sides show the data from the vITI and vSD schedules, respectively. The bar charts depict the observed data on the original scale, while the line charts depict the modelled reference-dependent effects as analysed data with the appropriate transformations. The line chart y-axes denote changes relative to the within-subject VEH measurement within the Latin square design. The zero value on the x-axis indicates the mean of the reference values for the whole cohort. Significant reference-dependent treatment effects are reflected by line slopes that significantly differ from 0. The line graphs include a shaded standard error phase, depicting the modelled standard error of the dose at X=0, modified by the standard error of the slope towards the edges. Abbreviations: *vSD*: variable stimulus duration, *vITI*: variable intertrial interval, *SCH*: SCH23390, *VEH*: vehicle, *FAR*: false alarm rate, *imp.*: impulsivity, *cons.*: conservative. N for vITI SCH: 36, vSD SCH: 35

**Fig. 2:**
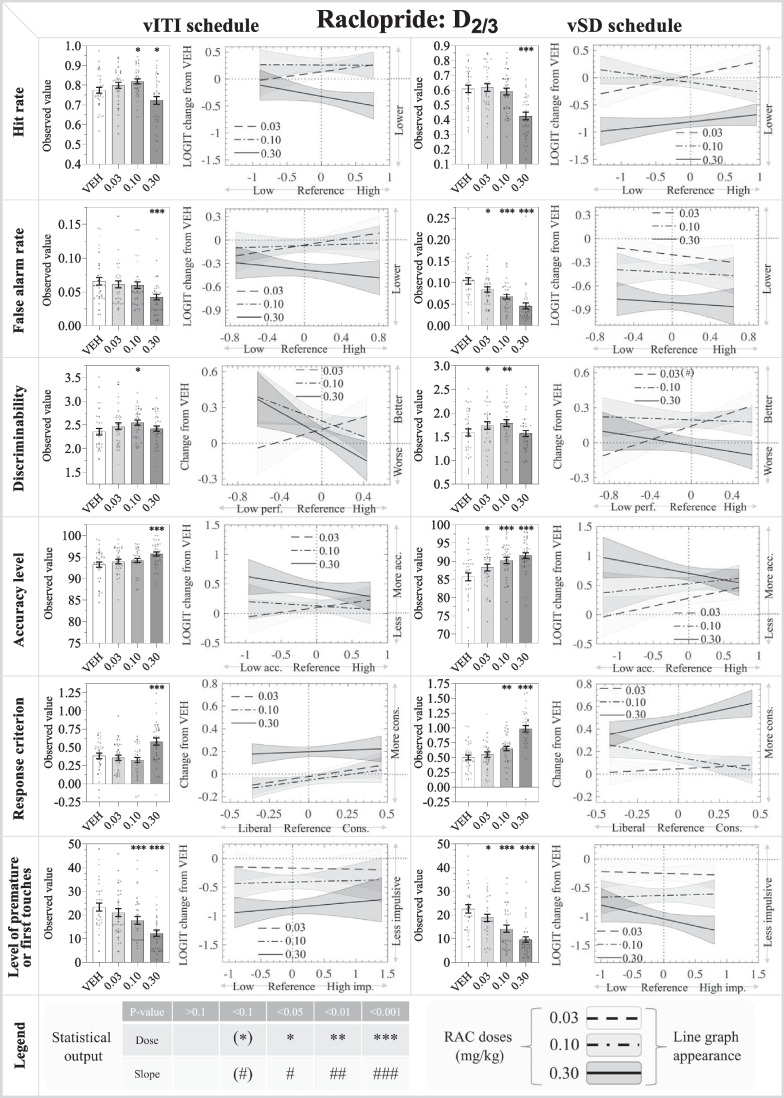
Results from the D_2/3_ receptor antagonist: raclopride (RAC: 0.03, 0.10, 0.30 mg/kg) in the rodent continuous performance test. The left- and right-hand sides show the data from the vITI and vSD schedules, respectively. The bar charts depict the observed data on the original scale, while the line charts depict the modelled reference-dependent effects as analysed data with the appropriate transformations. The line chart y-axes denote changes relative to the within-subject VEH measurement within the Latin square design. The zero value on the x-axis indicates the mean of the reference values for the whole cohort. Significant reference-dependent treatment effects are reflected by line slopes that significantly differ from 0. The line graphs include a shaded standard error phase, depicting the modelled standard error of the dose at X = 0, modified by the standard error of the slope towards the edges. Abbreviations: *vSD*: variable stimulus duration, *vITI*: variable intertrial interval, *SCH*: SCH23390, *VEH*: vehicle, *FAR*: false alarm rate, *imp.*: impulsivity, *cons.*: conservative. N for vITI RAC: 35, vSD RAC: 35

**Fig. 3:**
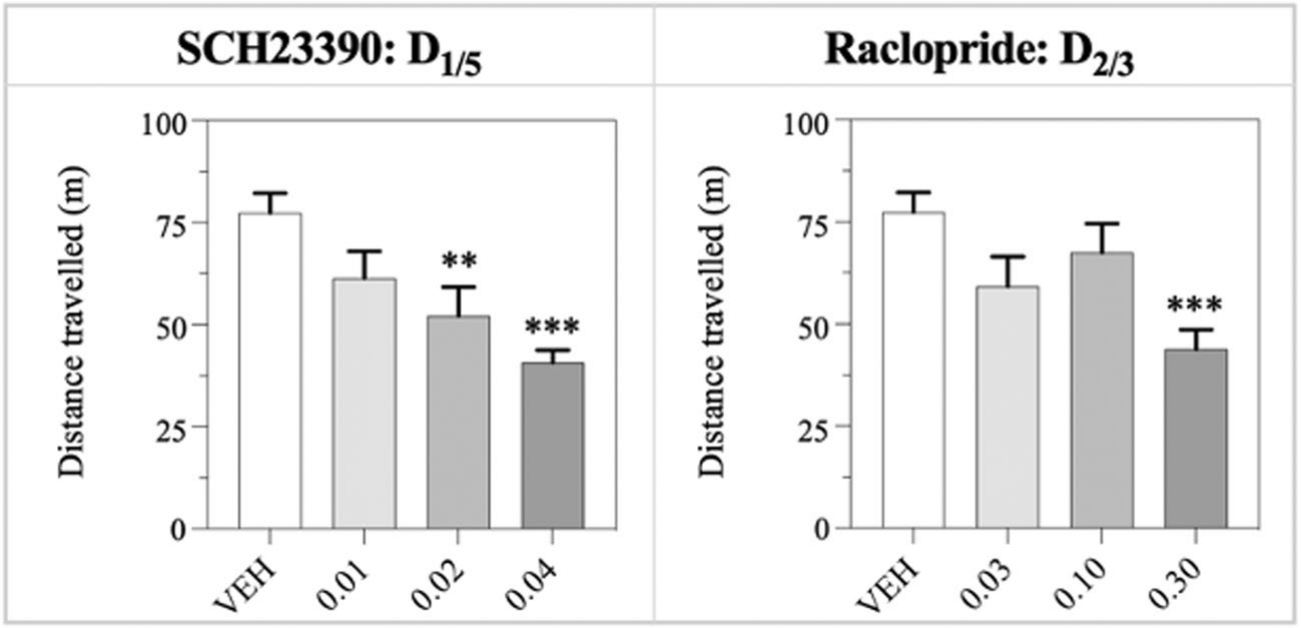
Total distance travelled in the locomotor assay, analysed through one-way ANOVA with multiple comparisons to the vehicle for the individual doses (Dunnett’s). The data was log-transformed prior to analysis and depicted as the non-transformed values (mean ± S.E.M.). The significance of individual doses is displayed, where trend values (0.05<*P*<0.1) are illustrated as (*), and significant values are illustrated as * / ** / *** for *P* < 0.05 / 0.01 / 0.001. *N* = 9–10 per dose

The results from the experiments with SCH and RAC are shown in Figs. 1 and 2, respectively. The results from both drugs in the rCPT are compiled in Table 2 for vITI data and Table 3 for vSD data, while the response latencies are shown in Table 4 for both schedules. The effects on locomotor activity are shown in Figure 3, and a summary of all the effects is provided in Table 5. Significant main and fixed effects of time and reference were found for most analyses, showing that reference values are strong predictors of outcome values, and that the performance of the mice drifted over time. These significant values confirm the importance of including reference and time values in the statistical modelling. The following sections describe the main effects of treatment, the post hoc analysis comparing the effects of each dose to the vehicle effects (including the fixed effects of dose and the dose:reference interaction), and the analysis of the response latencies. The significant values are described in detail, while the reader is referred to Tables 3 and 4 for further details pertaining to the non-significant results.

**Table 2:**
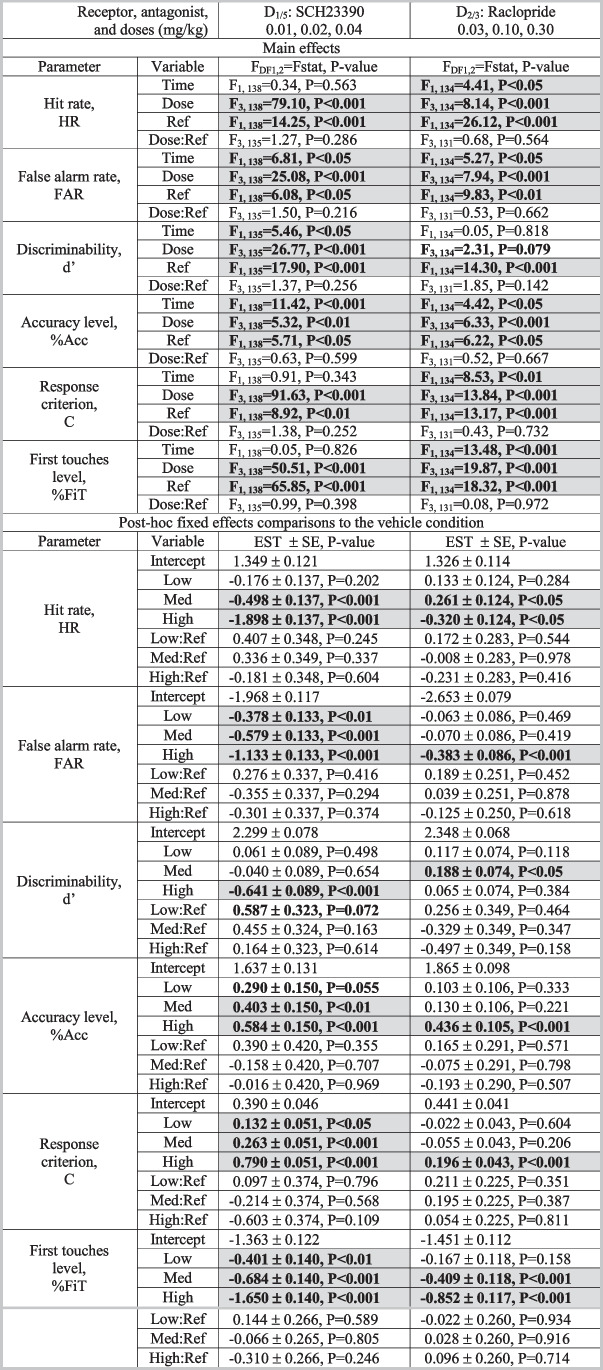
Statistical output of the mixed effects model for the variable intertrial interval (vITI) schedule. The results were analysed in a repeated measurements mixed effects model, using MATLAB version R2020b. The output is separated into the main effects from the model and those of the post-hoc fixed effects comparisons to the vehicle. We examined SCH23390 (0.01, 0.02, 0.04 mg/kg) and raclopride (0.03, 0.10, 0.30 mg/kg). Abbreviations: *DF*: degrees of freedom, *Fstat*: F statistic, *SE*: standard error of estimate, *Ref*: reference. Significant effects (P<0.05) are highlighted in grey and bold, and trend effects (0.05<P<0.1) are highlighted with bold font. N: vITI SCH: 36, vITI RAC: 35

**Table 3:**
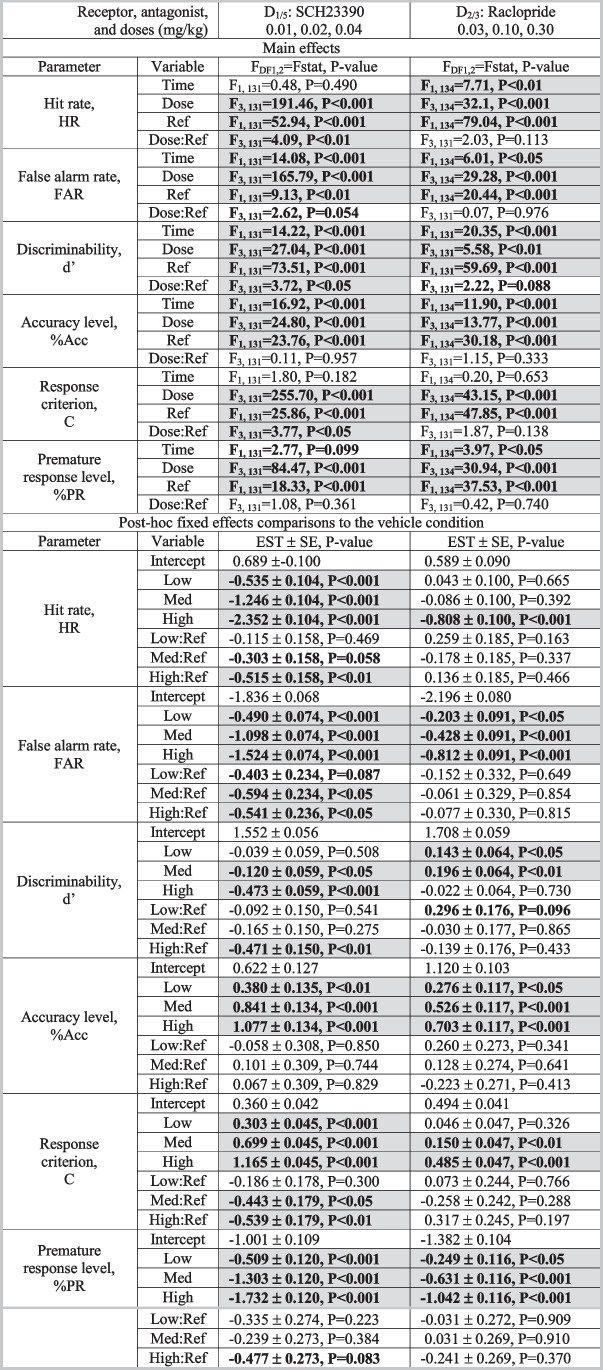
Statistical output of the mixed effects model for the variable stimulus duration (vSD) schedule. The results were analysed in a repeated measurements mixed effects model, using MATLAB version R2020b. The output is separated into the main effects from the model and those of the post-hoc fixed effects comparisons to the vehicle. We examined SCH23390 (0.01, 0.02, 0.04 mg/kg) and raclopride (0.03, 0.10, 0.30 mg/kg. Abbreviations: *DF*: degrees of freedom, *Fstat*: F statistic, *SE*: standard error of estimate, *Ref*: reference. Significant effects (P<0.05) are highlighted in grey and bold, and trend effects (0.05<P<0.1) are highlighted with bold font. N: vSD SCH: 35, vSD RAC: 35

**Table 4:**
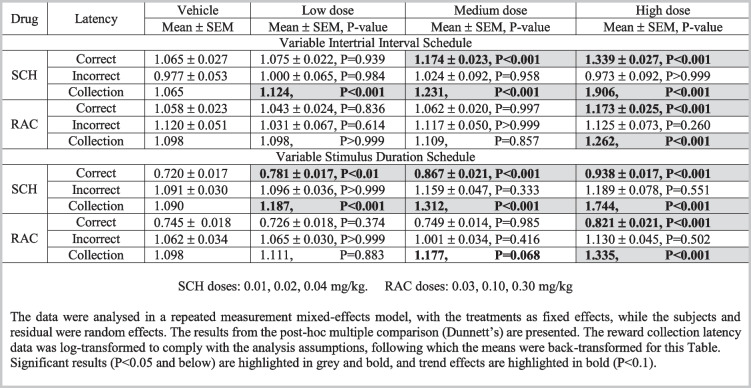
Overview of treatment effects on response latencies within the rCPT schedules. The results were analysed in a one-way analysis of variance with multiple comparisons to the vehicle (Dunnett), using Prism 9. Note, the reward collection latency values do not include SEM, as the means were log-transformed for the analysis and then back transformed for this table. Abbreviations: *rCPT*: rodent continuous performance test, *SEM*: standard error of the mean, *SCH*: SCH23390, *RAC*: raclopride. N: vITI SCH: 36, vSD SCH: 35, vITI RAC: 35, vSD RAC: 35

**Table 5:**
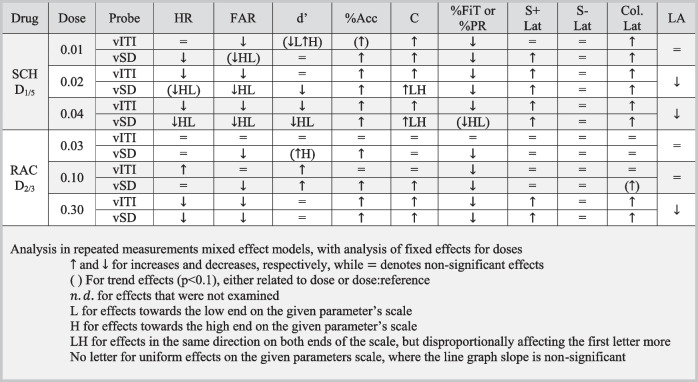
Overview of findings in the rCPT vSD and vITI schedules, and in the locomotor assay. Abbreviations: *rCPT*: rodent continuous performance task, *vSD*: variable stimulus duration schedule, *vITI*: variable intertrial interval schedule, *HR*: hit rate, *FAR*: False alarm rate, *d’*: discriminability, *%Acc*: accuracy level, *C*: response criterion, *%PR*: premature response level, *%FiT*: first touches level, *Lat*: latency, *Col.*: reward collection. N: vITI SCH: 36, vSD SCH: 35, vITI RAC: 35, vSD RAC: 35

### Variable Intertrial Interval Schedule

#### SCH23390

*Main effects:* There was a significant main effect of treatment on HR (F_3,138_ = 93.13; *P*<0.001), FAR (F_3,138_ = 25.08; *P*<0.001), d’ (F_3,138_ = 26.77; *P*<0.001), %Acc (F_3,138_ = 5.32; *P*<0.01), C (F_3,138_ = 91.63; *P*<0.001), and %FiT (F_3,138_ = 50.51; *P*<0.001).

*Specific dose effects:* HR was dose-dependently reduced by SCH, with significant reductions for 0.02 (*P*<0.001) and 0.04 mg/kg (*P*<0.001). FAR was significantly reduced by all SCH doses (*P*<0.01 for 0.01 mg/kg, *P*<0.001 for 0.02 and 0.04 mg/kg). These effects on HR and FAR were not reference-dependent. d’ was significantly reduced by 0.04 mg/kg SCH (*P*<0.001), indicating that SCH worsened discriminative performance. The 0.01 mg/kg dose showed a trend towards dose:reference interaction (*P*=0.072), where a positive slope indicates that 0.01 mg/kg SCH worsens performance in low-d’ mice and improved performance in high-d’ mice. %Acc was significantly increased by 0.02 (*P*<0.01) and 0.04 mg/kg SCH (*P*<0.001), and a trend effect was seen at 0.01 mg/kg (*P*=0.055). None of these dose effects were reference-dependent. Response criterion, C, was dose-dependently increased by SCH, indicating that SCH induced a more conservative response strategy (*P*<0.05 for 0.01 mg/kg, *P*<0.001 for 0.02 and 0.04 mg/kg). %FiT was dose-dependently reduced (*P*<0.01 for 0.01 mg/kg, *P*<0.001 for 0.02 and 0.04 mg/kg). None of the doses showed significant dose:reference interactions for the effects on %Acc, C, or %FiT, indicating that these effects were not reference-dependent.

*Latency effects:* SCH significantly increased correct latency at 0.02 (*P*<0.001) and 0.04 mg/kg (*P*<0.001), whereas there were no significant effects on the incorrect latency at any of the tested doses. All three SCH doses significantly increased reward collection latency (*P*<0.001).

#### Raclopride

*Main effects:* There was a significant main effect of treatment on HR (F_3, 134_ = 8.14; *P*<0.001), FAR (F_3,134_ = 7.94; *P*<0.001), %Acc (F_3,134_ = 6.33; *P*<0.001), C (F_3,134_ = 13.84; *P*<0.001), and %FiT (F_3,134_ = 19.87; *P*<0.001), and a trend towards a main effect on d’ (F_3,134_ = 2.31; *P*=0.079).

*Specific dose effects:* HR was significantly increased by 0.10 mg/kg RAC (*P*<0.05), but significantly reduced by 0.30 mg/kg RAC (*P*<0.05). FAR was significantly reduced by 0.30 mg/kg RAC (*P*<0.001). d’ was increased by 0.10 mg/kg RAC (*P*<0.05). %Acc and C were both significantly increased by 0.30 mg/kg RAC (*P*<0.001 for both). %FiT was significantly reduced by 0.10 and 0.30 mg/kg RAC (*P*<0.001 for both). None of these effects were reference-dependent.

*Latency effects:* At 0.30 mg/kg, RAC increased correct latency and reward collection latency (*P*<0.001 for both), but did not significantly affect incorrect latency.

### Variable stimulus duration schedule

#### SCH23390

*Main effects:* There was a significant main effect of treatment on all parameters (HR: F_3,131_ = 191.46; *P*<0.001) (FAR: F_3,131_ = 165.79; *P*<0.001) (d’: F_3,131_ = 27.04; *P*<0.001) (%Acc: F_3,131_ = 24.80; *P*<0.001) (C: F_3,131_ = 255.70; *P*<0.001) (%PR: F_3,131_ = 84.47; *P*<0.001).

*Specific dose effects:* HR was dose-dependently reduced by SCH (*P*<0.001 for all doses). The dose:reference interaction was near-significant for the 0.02 mg/kg dose (*P*=0.058) and significant for the 0.04 mg/kg dose (*P*<0.01), which showed a stronger effect in high-HR mice. FAR was significantly and dose-dependently reduced by all SCH doses (*P*<0.001), and the 0.01 mg/kg dose showed a trend dose:reference interaction (*P*=0.087), while both 0.02 and 0.04 mg/kg showed significant dose:reference interactions (*P*<0.05 for both). All doses reduced FAR more prominently in high-FAR mice. d’ was significantly reduced by 0.02 (*P*<0.05) and 0.04 mg/kg SCH (*P*<0.001). The 0.01 mg/kg dose showed a significant dose:reference interaction (*P*<0.01), worsening performance more prominently in high-d’ mice. %Acc was dose-dependently increased by SCH (*P*<0.01 for 0.01 mg/kg, *P*<0.001 for 0.02 and 0.04 mg/kg), but these effects were not reference-dependent. C was dose-dependently increased by SCH (*P*<0.001 for all doses). The 0.02 (*P*<0.05) and 0.04 mg/kg doses (*P*<0.01) showed significant dose:reference interactions, generally increasing C, but mostly in low-C mice. %PR was dose-dependently decreased by SCH (*P*<0.001 all doses), and the 0.04 mg/kg dose showed a trend towards a reference-dependent effect (*P*=0.083), decreasing impulsivity mostly in high-%PR mice.

*Latency effects:* All SCH doses increased correct latency (*P*<0.01 for 0.01 mg/kg, *P*<0.001 for 0.02 and 0.04 mg/kg) and reward collection latency (*P*<0.001 for all), while none of the doses significantly changed incorrect latency.

#### Raclopride

*Main effects:* There were significant main effects of treatment on all parameters (HR: F_3,134_ = 32.11; *P*<0.001) (FAR: F_3,134_ = 29.28; *P*<0.001) (d’: F_3,131_ = 5.58; *P*<0.01) (%Acc: F_3,134_ = 13.77; *P*<0.001) (C: F_3,134_ = 43.15; *P*<0.001) (%PR: F_3,134_ = 30.94; *P*<0.001).

*Specific dose effects*: HR was significantly reduced by 0.30 mg/kg RAC (*P*<0.001). FAR was dose-dependently reduced by RAC (*P*<0.05 for 0.03 mg/kg, and *P*<0.001 for 0.10 and 0.30 mg/kg). These effects were not reference-dependent. d’ was significantly increased by 0.03 (*P*<0.05) and 0.10 mg/kg RAC (*P*<0.01). The 0.03 mg/kg dose showed a trend dose:reference interaction (*P*=0.096), increasing d’ more prominently in high-d’ mice. %Acc was dose-dependently increased by RAC (*P*<0.05 for 0.03 mg/kg, *P*<0.001 for 0.10 and 0.30 mg/kg). C was significantly increased by 0.10 (*P*<0.01) and 0.30 mg/kg (*P*<0.001) RAC. %PR was dose-dependently decreased by RAC (*P*<0.05 for 0.03 mg/kg, *P*<0.001 for 0.10 and 0.30 mg/kg). These effects were not reference-dependent.

*Latency effects:* At 0.30 mg/kg, RAC increased correct latency (*P*<0.001), 0.10 mg/kg trended to increase (*P*=0.068), and 0.30 mg/kg (*P*<0.001) significantly increased, reward collection latency, while no effects were found on incorrect latency.

### Locomotor activity

The total distance travelled is shown in Figure 3.

*SCH23390:* The one-way ANOVA showed a significant main effect of treatment on distance travelled (F_3,34_ = 7.64; *P*<0.001). Post hoc Dunnett’s test showed that SCH dose-dependently decreased total distance travelled, with significant effects of 0.02 mg/kg (*P*<0.01) and 0.04 mg/kg (*P*<0.001).

*Raclopride* ***:*** The one-way ANOVA showed a significant main effect of treatment on locomotor activity (F_3,33_ = 5.67; *P*<0.01). Post hoc Dunnett’s test showed a significant decrease at 0.30 mg/kg (*P*<0.001).

### Data summary

Table 5 gives an overview of all data.

## Discussion

We used DA R antagonism in the vSD and vITI rCPT schedules to examine the effects of reducing receptor activity on different behaviours measured. The effects on each parameter were related to the reference levels of the mice. We also calculated response accuracy to increase the comparability to literature results from the 5-CSRTT. The following sections begin with a summary of the treatment effects, followed by a specific discussion of the effects on attention- and impulsivity-related measures, and conclude with effects on response latencies and locomotion. In addition to affecting measures of attention and impulsivity, some of the drug doses examined in this current paper and in the adjoining paper examining NA adrenoceptor antagonists (Klem et al. 2023) also showed effects on measures of motor activity and motivation (locomotor activity, response and reward collection latencies). Both attention and impulsive responses are partly dependent on motivation, and, similar to other operant tasks, the rCPT relies on motor responses. Therefore, any treatment effects on locomotor activity and/or response/reward collection latencies should therefore be considered when interpreting treatment effects on measures of attention or impulsivity.

### D_1/5_ R antagonism reduced responding, impulsivity, and locomotor activity, and caused mixed effects on attentional performance in both schedules

SCH reduced responding in both schedules, as measured by reductions in HR and FAR and an increase in C. The effects on attentional performance were mixed in both schedules, as SCH increased %Acc and decreased d’. SCH reduced %FiT and %PR. The effects were generally similar in the two schedules, but reference-dependent effects were seen in the vITI schedule, where SCH had a stronger effect in mice with high responding (low C value), high d’, and high accuracy. SCH increased latencies of both correct responses and reward collection, without affecting incorrect response latency. SCH also significantly reduced locomotor activity.

If we assume that the relation between D_1_-like receptor activity and rCPT performance follows an inverted U-shaped pattern (Gamo et al. 2010), D_1/5_ R antagonism would be expected to impair performance in animals with low DA levels and improve in animals with high DA levels. The SCH-induced reduction in d’, particularly in mice with a high reference d’ values in the vITI schedule, is in line with such a U-shaped relationship. Interpreting the effects on attentional performance is complicated by the finding that SCH also improved accuracy, which appears to contradict the dose-dependent reduction in d’. An important difference between d’ and accuracy is that misses (omissions) affect d’, but not accuracy, since accuracy only considers active responses. Furthermore, d’ and accuracy also differ by use of Z-scores in the d’ calculation. Since SCH caused a marked reduction in overall responsiveness (increased C), the increased number of misses is reflected by a decrease in d’. However, the reduced number of responses apparently reduced FAR proportionally more than HR, leading to increased accuracy. Including both d’ and accuracy nuances the understanding of treatment effects on attentional performance. Our findings comply with a 5-CSRTT rat study, where systemic SCH increased both accuracy and omissions, effects that were related to parallel reductions in HR and FAR (Van Gaalen et al. 2006). Our observation is also in line with 5-CSRTT studies in rats examining local administration in the nucleus accumbens (NAc), where SCH improved accuracy through actions in the NAc core and increases omissions through the NAc shell region (Pattij et al. 2007). A 5-CSRTT rat study showed inverted U-shaped dose-response effects of both SCH and a D_1/5_ receptor agonist, when given locally in the NAc (Pezze et al. 2007). Collectively, these results suggest a non-linear relationship between the level of D_1/5_ R activity and accuracy.

In a rat 5-CSRTT study, administration of SCH or a D_1/5_ R agonist in the medial prefrontal cortex improved accuracy and slightly increased responsiveness (decreased omissions) in low-performers, while antagonism with SCH impaired accuracy without affecting omissions in high performers (Granon et al. 2000). In another rat 5-CSRTT study, systemic D_1/5_ R agonism made rats less responsive without affecting accuracy (Winstanley et al. 2010). A recent rat 5-CSRTT study complicated the matter further, as accuracy was worsened by SCH at the short ITI (3s) but improved at the longer ITI (7s) (Balachandran et al. 2018). The authors suggested that opposite effects result from differences in attentional demand, where high phasic PFC catecholamine transmission during longer ITI could be dampened to more optimal levels by D_1/5_ R antagonism, which aligns with an inverted U-shaped relationship.

Our findings of reduced impulsivity (%FiT and %PR) are in accordance with rat 5-CSRTT studies where systemic SCH administration also decreased %PR (Van Gaalen et al. 2006; Balachandran et al. 2018). Intra-NAc administration of SCH reduced %PR in one study (Pattij et al. 2007). In another study, %PR was not significantly affected by SCH, while a D_1/5_ R agonist increased %PR (Pezze et al. 2007), consistent with the SCH-induced %PR decrease observed in the present study. D_1/5_ R agonism has also been reported to reduce %PR in the rat 5-CSRTT (Winstanley et al. 2010). Considering that SCH consistently reduces PR in the literature, it is interesting that similar effects have been observed with D_1/5_ R agonism. Overall, the present and previous findings corroborate the hypothesis that D_1/5_ R activity regulates attentional performance and impulsivity in a non-linear manner, seemingly conforming to a U-shaped relationship, and depending on task demands.

The 0.02 and 0.04 mg/kg SCH doses reduced LA and increased response and reward collection latencies, consistent with the well-known role of D_1_ R in the direct motor pathway of the basal ganglia (Singh-Bains et al. 2016; Pretegiani and Optican 2017). In rat 5-CSRTT studies, SCH showed no effect on response latencies following systemic (Van Gaalen et al. 2006) or local administration in the NAc or OFC, but a D_1/5_ agonist slowed both correct responses and reward collection (Pattij et al. 2007; Winstanley et al. 2010). At 0.1 mg/kg, SCH did not significantly affect locomotor activity in the spontaneously hypertensive rat model of ADHD (Umehara et al. 2013). Doses equal to or exceeding 0.05 mg/kg SCH reduced locomotor activity of coloboma mice, and 0.2 mg/kg showed significant reductions in control mice (Fan and Hess 2008). Based on these studies, reductions in locomotor activity are expected, albeit not at the SCH dose range used in the present study.

### D_2/3_ R antagonism reduced responding, improved attentional performance in both rCPT schedules, and reduces locomotor activity at higher doses

HR was decreased by the highest RAC dose (0.3 mg/kg) in both schedules, while the low 0.03 mg/kg dose increased HR in the vITI schedule. RAC reduced FAR in both schedules, with the strongest effect in the vSD schedule. These effects led to improvements of d’ in both schedules: the improvement seen in the vITI schedule was driven by a HR increase and a modest FAR decrease, while the improvement in the vSD schedule was driven by the relatively stronger reduction in FAR than HR. RAC also improved %Acc in both schedules, showing the strongest effects in the vSD schedule. RAC reduced impulsivity in both schedules and generally reduced overall responding. RAC slowed both correct responses and reward collection and decreased locomotor activity, indicating reduced motivation and motoric activity.

Most studies examining the role of DA in the context of an inverted U-shaped relationship often focus on D_1/5_ R signalling, showing that low-to-moderate signalling improves performance, while excessive D_1/5_ R activity impairs performance (Gamo et al. 2010). DA has a higher affinity for D_2/3_ Rs than for D_1/5_ Rs, and this might also contribute to an inverted U-shaped relationship between DA levels and attention (Marcellino et al. 2012; Hunger et al. 2020). A rat 5-CSRTT study showed a tendency towards inverted U-shaped pattern on attentional performance following intra-NAc D_2/3_ R antagonism (Pezze et al. 2007). In addition to differential involvement of of D_1/5_-and D_2/3_ in attention regulation, U-shaped patterns may also arise from differential engagement of D_2/3_ autoreceptors and post-synaptic D_2/3_ receptors. One study showed a non-linear (triphasic) effects on reversal learning performance following intra-caudate D_2/3_ R agonism (Horst et al. 2019). The authors suggested that the lower doses of the agonist worsened performance by preferably engaging D_2_ autoreceptors, while the moderate doses improved performance by optimally engaging both the D_2_ autoreceptors and post-synaptic D_2/3_ Rs, and that the higher doses worsened performance by excessive activation of the post-synaptic D_2/3_ Rs (Horst et al. 2019). We speculate that our observed biphasic pattern following RAC treatment relates to this study. Rat 5-CSRTT studies examining D_2/3_ R antagonism have shown decreased accuracy (Balachandran et al. 2018), or no effect on accuracy (Van Gaalen et al. 2006), and increased omissions following systemic (Van Gaalen et al. 2006) or intra-NAc administration (Pattij et al. 2007). These effects on accuracy oppose our findings, while the effects on omissions partially support our observations at higher doses. Our finding of an antagonist-induced increase in HR in the vITI schedule, corresponding to decreased omissions, is in line with reports that D_2/3_ R agonism increased omissions in the rat 5-CSRTT (Winstanley et al. 2010; Fernando et al. 2012). Increased omissions are also observed following both agonism and antagonism of D_2/3_ Rs in a rat 5-CSRTT study following systemic administration, but neither agonism nor antagonism changed accuracy (Besson et al. 2010). Similarly, in the stop signal reaction time task (SSRT), both D_3_ R agonism and antagonism decreased go accuracy, equivalent to decreased HR in the rCPT, while a D_2/3_ R antagonist showed no significant effect on HR (Bari and Robbins 2013). The results of these experiments are relevant for antipsychotic medication, as they involve antagonism of D_2/3_ Rs, which alleviates positive symptoms of schizophrenia disorder, but shows limited effects on cognitive symptoms (Macpherson and Hikida 2019). Our results lend some support to pro-attentive effects of D_2/3_ R antagonism at the lower and medium RAC doses.

The RAC-induced decreases in %FiT and %PR are in contrast to several 5-CSRTT studies in rats and mice, where systemic D_2/3_ R antagonism showed no effect on %PR (Van Gaalen et al. 2006; Besson et al. 2010; Balachandran et al. 2018), and where D_2/3_ R and D_2_ R agonism decreased %PR (Besson et al. 2010; Winstanley et al. 2010; Fernando et al. 2012). Notably, local NAc administration of a D_2/3_ R antagonist in high-impulsive rats increased %PR in the 5-CSRTT when administered in the NAc shell, and reduced %PR when administered in the NAc core (Besson et al. 2010), although a similar study did not report effects of D_2/3_ R antagonism in the NAc shell or core on %PR (Pattij et al. 2007). Regardless of the complex directions of effects, robust correlations have been found in humans between striatal D_2/3_ R availability and trait impulsiveness during resting state (e.g. Anderson et al. 2017).

Our findings that RAC increased latencies to correct responses and reward collection are in accordance with several rat 5-CSRTT studies, where D_2/3_ R antagonism slowed general and correct responses (Van Gaalen et al. 2006; Pattij et al. 2007; Besson et al. 2010). However, this interpretation is complicated by reports that systemic D_2/3_ R agonism also increase correct and reward collection latencies across different rat 5-CSRTT studies (Besson et al. 2010; Winstanley et al. 2010; Fernando et al. 2012). D_2/3_ R and subtype-selective manipulations have been investigated in the SSRT, where D_2_ R antagonism showed no effects, while D_3_ R agonism slowed mean reaction times, and D_3_ R antagonism slowed both stop signal and mean reaction times (Bari and Robbins, 2013). The slower response speeds may relate to the locomotor-depressant effects observed at 0.3 mg/kg RAC in the present study. The motor-depressive effect of the high dose is in line with a previous report, where RAC doses equal to or exceeding 0.3 mg/kg reduced activity for coloboma mice, while similar depressant effects were observed at doses equal to or above 0.1 mg/kg for control mice (Fan and Hess 2008).

## Conclusion

This study characterised the role of DA R antagonists in the rCPT. DA R antagonism showed significant effects on most rCPT parameters, indicating that DA is a key regulator of rCPT behaviours. Our results suggest that endogenous DA increases overall responding through D_1/5_ and D_2/3_ Rs, and that D_2/3_ Rs may also inhibit responding under certain conditions (HR for RAC in the vITI schedule). The antagonist results also indicate that effects of DA on discriminability depends on receptor subtype, improving via D_1/5_ Rs and worsening performance via D_2/3_ Rs, and that activity of either of these subtypes worsens accuracy. Further, DA may increase waiting impulsivity through actions on both D_1/5_ Rs and D_2/3_ Rs. Finally, the results indicate that DA hastened correct and reward collection responses through both D_1/5_ and D_2/3_ Rs, and that this corresponds to a general motor-stimulant effect of DA. The clear effects on locomotor activity and response latencies suggest that changes in motivation and/or locomotion contributes to the effects on attention and impulsivity.

A secondary aim of this study was to compare the effects in the vITI and vSD schedules. The vSD schedule was expected to be more sensitive to effects on attention, and the vITI schedule more sensitive to effects on impulsivity, but the results obtained in the two schedules show largely overlapping effects. Further optimization of the schedules is required to characterize possible differences in sensitivity towards effects on attention or impulsivity.

### **Supplementary information**

ESM 1
