# Supplementary material for: Assessing attention and impulsivity in the variable stimulus duration and variable intertrial interval rodent continuous performance test schedules using dopamine receptor antagonists in female C57BL/6JRj mice
Source: Psychopharmacology (Berl). 2023 Jun 28;240(8):1651–66. doi: 10.1007/s00213-023-06387-7 (PMC10349733; doi:10.1007/s00213-023-06387-7)
Supplement: Supplementary file 1 — ESM 1 [file 213_2023_6387_MOESM1_ESM.docx]

**Supplementary Material**

**The FoT/FiT ratio**

We have previously modified the analysis protocol for a rCPT vITI schedule, which led to the development of the parameters: %FiT and FoT/FiT (Prichardt et al. 2023). Our study indicated FiT and FoT responses were unrelated behaviours, as the two parameters were uncorrelated and were differentially sensitive to catecholamine manipulation. In this previous study, amphetamine reduced %FiT and increased the FoT/FiT ratio, while atomoxetine reduced %FiT but did not affect the FoT/FiT ratio (Prichardt et al. 2023). We also observed differential effects on the two parameters in our study examining noradrenaline adrenoceptor antagonism in the rCPT vITI schedule (Klem et al. 2023). In this study, doxazosin (α_1_) reduced %FiT and increased FoT/FiT, yohimbine (α_2_) increased %FiT, but did not affect FoT/FiT, while propranolol (β_1/2_) increased both %FiT and the FoT/FiT ratio (Klem et al. 2023). These combined results indicate the rCPT %FiT parameter provides a more sensitive measure of waiting impulsivity relative to the initial rCPT %PR, but this remains to be validated by future studies. Moreover, since 5-CSRTT FiTs trigger a timeout period as a punishment, during which FoTs are not recorded, the rCPT %FiT parameter should more closely resemble 5-CSRTT %PR (Bari et al. 2008, Prichardt et al. 2023). We our current FoT/FiT results to support characterisation of the parameter but refrain from speculating on the nature of the behaviour. The FoT/FiT ratio is calculated based on the following:

Following touches per first touch: $\frac{FoT}{FiT}=\frac{Centre touches during 12s ITI restart loops occuring within 0-0.5 s}{Initial centre touches during 12s ITI}$

The FoT/FiT ratio was not restricted by ceiling effects, but still required transformation to comply with the model assumptions, hence the results were transformed using the natural logarithm (ln). The FoT/FiT ratio results are presented in Appendix Figure 4 and in Table 6. The following sections describe the main effects of treatment and the post-hoc analysis of the individual antagonist doses on the FoT/FiT ratio in the vITI schedule. For brevity, only trend or significant main and fixed effects will be described in detail.

*SCH23390:* There was not a significant main effect of treatment on the FoT/FiT ratio. Post-hoc analysis of the effect of each dose compared to the vehicle showed no significant effects on the FoT/FiT ratio, nor were the effects reference-dependent.

*Raclopride:* There was not a significant main effect of treatment on the FoT/FiT ratio. None of the RAC doses showed significant dose fixed effects. At 0.30 mg/kg, RAC showed a significant dose:reference interaction, increasing the FoT/FiT ratio for high-FoT/FiT mice and reducing the parameter for mice with low reference values.

Both SCH and RAC reduced %FiT but did not show prominent effects on the FoT/FiT ratio in the vITI schedule. These results indicate that FoT behaviour is less sensitive to endogenous dopamine receptor tonus, relative to the more prominent effects we observed with antagonism of noradrenaline adrenoceptors (Klem et al. 2023).

**Table 6** *Statistical output of the mixed effects model for following to first touches ratio (FoT/FiT) in the the variable intertrial interval (vITI) schedule. The results were analysed in a repeated measures mixed effects model, using MATLAB version R2020b. The output is separated into the main effects from the model and those of the post-hoc fixed effects comparisons to the vehicle. We examined doxazosin (1, 3, 10 mg/kg), yohimbine (0.1, 0.3, 1.0 mg/kg), and propranolol (1, 3, 10 mg/kg). Low, medium, and high refer to the relative concentrations of the drug doses. Significant effects (P<0.05) are highlighted with grey. Trend-effects (0.05<P<0.1) are highlighted with light grey. Abbreviations: DF: degrees of freedom, Fstat: F statistic, SE: standard error of estimate, Ref: reference. N: 36*

| Receptor, antagonist,  and doses (mg/kg) | | D_1/5_: SCH23390  0.01, 0.02, 0.04 | D_2/3_: Raclopride  0.03, 0.10, 0.30 |
| --- | --- | --- | --- |
| Main effects | | | |
| Parameter | Variable | F_DF1,2_=Fstat, P-value | F_DF1,2_=Fstat, P-value |
| Following to first touches ratio, FoT/FiT | Time | F_1, 133_=1.52, P=0.220 | F_1, 131_=0.44, P=0.509 |
|  | Dose | F_3, 133_=0.95, P=0.418 | F_3, 131_=0.83, P=0.481 |
|  | Ref | F_1, 133_=71.55, P<0.001 | F_1, 131_=94.19, P<0.001 |
|  | Dose:Ref | F_3, 130_=1.21, P=0.307 | F_3, 131_=4.02, P<0.01 |
| Post-hoc fixed effects comparisons to the vehicle condition | | | |
| Parameter | Variable | EST ± SE, P-value | EST ± SE, P-value |
| Following to first touches level, FoT/FiT | Intercept | 1.270 ± 0.080 | 1.405 ± 0.067 |
|  | Low | 0.047 ± 0.085, P=0.578 | 0.058 ± 0.075, P=0.443 |
|  | Med | 0.019 ± 0.085, P=0.828 | -0.060 ± 0.075, P=0.426 |
|  | High | 0.138 ± 0.088, P=0.118 | 0.008 ± 0.075, P=0.918 |
|  | Low:Ref | -0.178 ± 0.228, P=0.435 | 0.077 ± 0.196, P=0.697 |
|  | Med:Ref | -0.093 ± 0.230, P=0.687 | -0.246 ± 0.196, P=0.212 |
|  | High:Ref | 0.250 ± 0.236, P=0.290 | 0.424 ± 0.197, P<0.05 |


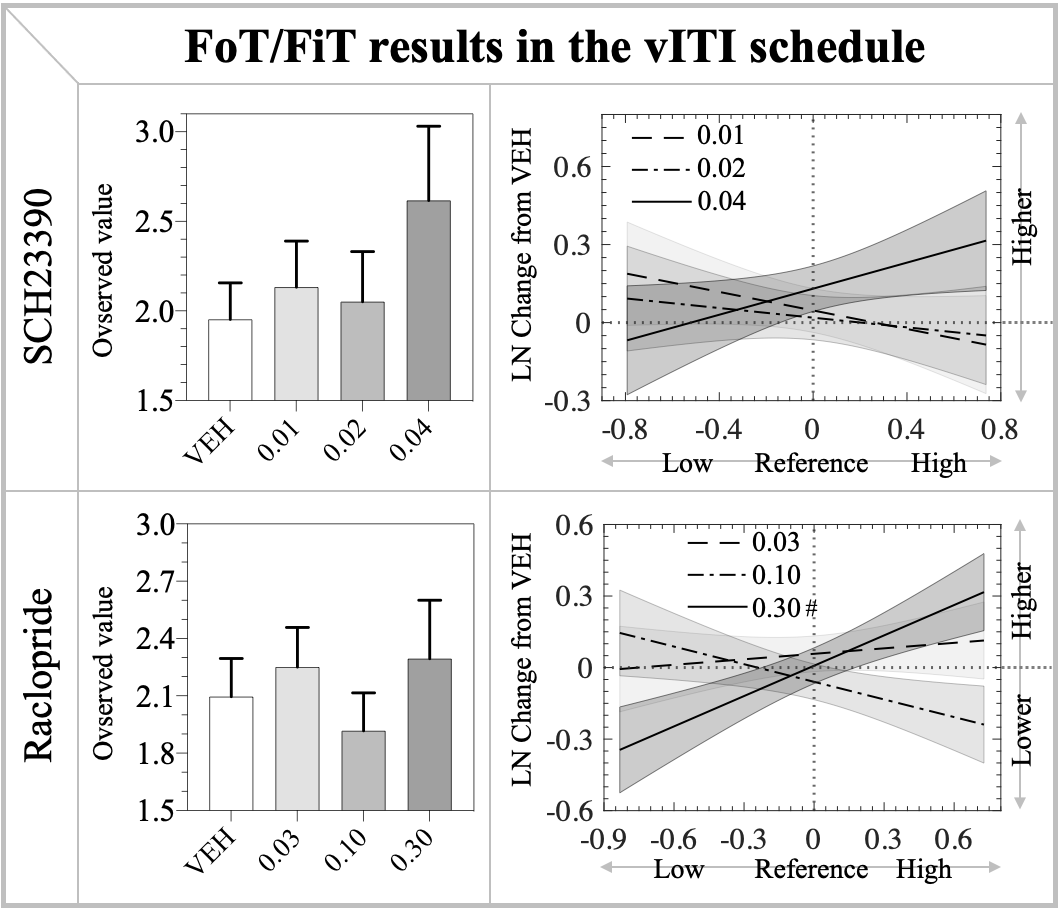


**Fig. 4** *Results from the D_1/5_ receptor antagonist: SCH23390 (SCH: 0.01, 0.02, 0.04 mg/kg) and the D_2/3_ receptor antagonist: raclopride (RAC: 0.03, 0.10, 0.30 mg/kg) in the rodent continuous performance test variable intertrial interval (vITI) schedule. The bar charts depict the observed data, while the line graphs depict the analysed data with the appropriate transformations. The line graph y-axes denote changes from the vehicle (VEH) measurement within the Latin square design. The line graph x-axes arrange the mice according to their average flanking vehicle measurements obtained outside of the Latin square design. Significant reference-dependent effects are shown as significant line graph slopes. The line graphs include a shaded standard error phase, depicting the standard error of the mean for the dose as the phase height at X=0, which is modified by the standard error of the slope towards the edges of the line. N: 36*
